# *Listeria monocytogenes* Isolates from Meat Products and Processing Environment in Poland Are Sensitive to Commonly Used Antibiotics, with Rare Cases of Reduced Sensitivity to Ciprofloxacin

**DOI:** 10.3390/life13030821

**Published:** 2023-03-17

**Authors:** Iwona Kawacka, Bernadeta Pietrzak, Marcin Schmidt, Agnieszka Olejnik-Schmidt

**Affiliations:** Department of Food Biotechnology and Microbiology, Poznan University of Life Sciences, Wojska Polskiego 48, 60-627 Poznan, Poland

**Keywords:** food safety, food pathogens, antibiotics, biodiversity, fingerprinting

## Abstract

Antibiotic resistance is a global health problem, causing not only an increased mortality rate of bacterial infections but also economic losses due to, among other reasons, the need for longer hospital stays. *Listeria monocytogenes* is one of the foodborne pathogens with the ability to induce a serious illness called listeriosis, with approximately 20–30% fatal outcomes. The treatment regimen of listeriosis in humans includes the administration of antibiotics (in most cases, ampicillin or trimethoprim with sulfamethoxazole in case of allergies to β-lactams), so the resistance of this pathogen to antibiotics can potentially lead to increased mortality. The antibiotic sensitivity status of *n* = 153 *L. monocytogenes* isolates originating from meat food samples (raw and processed) and meat-processing environment (both contacting and non-contacting with food) collected between October 2020 and November 2021 in Poland was examined in this study. Susceptibility to antibiotics was determined using the disc diffusion method on Mueller–Hinton agar plates. All collected samples were susceptible to 9 antibiotics: ampicillin (10 µg), chloramphenicol (30 µg), erythromycin (15 µg), gentamicin (10 µg), penicillin (10 IU), streptomycin (10 µg), sulfamethoxazole/trimethoprim (1.25/23.75 µg), tetracycline (30 µg) and vancomycin (30 µg). Some of the isolates (n = 10; 6.5%) showed reduced susceptibility to ciprofloxacin (5 µg), which was classified as an intermediate response. All these ten isolates were collected from surfaces contacting with food in food-processing facilities.

## 1. Introduction

### 1.1. Antibiotics, Antibiotic Resistance

Antimicrobials are medicines used to prevent and treat infections in humans, animals, and plants. Antibiotics are one of the types of antimicrobials (among antivirals, antifungals, and antiparasitics) used specifically to prevent or treat bacterial infections [1]. World Health Organization, Geneva, Switzerland (WHO) states that antimicrobial resistance (AMR) is a global health and development threat and a factor of significant economic losses. Not only is AMR a reason for death and disability in affected people, but it also creates the need for longer hospital stays and access to more expensive medicines [1]. In general, antibiotic resistance is a natural phenomenon that occurs when bacteria are exposed to antibiotics, which causes selective pressure. Susceptible bacteria are killed or inhibited, whereas resistant bacteria have greater chances of surviving [2]. Factors responsible for the rapid rise of resistant infections include poor public health infrastructure and a high burden of disease, widespread antibiotic use in animal farming, and the unregulated sale of cheap antibiotics [3].

According to some authors, undertaking a coordinated and comprehensive multinational response to address AMR is critical [4]. Recent systematic analysis of antibiotic resistance indicated that AMR is an issue in all regions of the world and, as a health problem, it is as large as HIV or malaria, potentially even greater [5]. It was estimated that by the year 2050, as many as 10 million people will die each year due to the rise of drug-resistant infections if appropriate actions are not taken [6]. Although this particular estimation has been quoted repeatedly and has reached more than 3000 citations [7], it was also criticized for insufficient scientific data and inaccuracy [8]. However, critics still claim that the AMR burden is likely to increase over time and that urgent action to overcome AMR is required [8]. Proposed solutions aiming to tackle AMR include: raising global awareness, improving hygiene to prevent the spread of infections, reducing unnecessary usage of antimicrobials, improving global surveillance of drug resistance, promoting rapid diagnostic to cut unnecessary use of antibiotics, promoting development and use of vaccines, improving the numbers and salaries of the people working with infectious diseases, establishing a Global Innovation Fund for early-stage and non-commercial research, promoting investment in new drugs, as well as improving existing ones and building a global coalition for real action [1,6,9,10,11]. A reduction of nontherapeutic use of antibiotics in animal agriculture is also crucial [12], as there is a direct link between antibiotic use in farms and the spread of antibiotic resistance to human populations [13]. What is more, the practice of using antibiotic formulations licensed for humans in animals should also be addressed in order to prevent the spread of antibiotic resistance between humans and animals [14]. 

### 1.2. Listeria monocytogenes and Food Safety

*Listeria monocytogenes* is a rod-shaped Gram-positive bacterium common in the environment; found in water, soil, and feces. Due to its ubiquitous nature, the existence of this bacterium in processing environments can occur, in some cases leading to bacterial contamination of final food products. *L. monocytogenes* can endure various stresses, such as sanitizers and pH. It can proliferate in refrigeration conditions, as well as survive mild heating (45 °C). In humans, after ingestion of contaminated foods, *L. monocytogenes* can cause life-threatening infections with symptoms such as meningitis, encephalitis, spontaneous abortion, or miscarriage due to its ability to cross the intestinal barrier, the blood–brain barrier, and the fetoplacental barrier. Usually, healthy individuals are not likely to experience severe infections. However, people with compromised immune systems (including the elderly, newborns, and pregnant women) are especially prone to life-threatening outcomes. *L. monocytogenes* is one of the leading causes of death from foodborne pathogens [15,16,17]. Many sources state that the mortality rate of listeriosis falls between 20% to 30% [17,18,19,20,21,22]. In the recent prospective observational cohort study, which included 818 cases from 372 centers, authors found that the overall 3-month mortality was 46% for bacteremia and 30% for neurolisteriosis, with only 39% of patients with neurolisteriosis making a full recovery [23].

*L. monocytogenes* is an organism under the surveillance of institutions such as the Centers for Disease Control and Prevention, Atlanta, Georgia state U.S. (CDC) [24] and European Centre for Disease Control and Prevention, Solna, Sweden (ECDC) [25]. The bacterium caused 2183 cases of illness in the European Union (EU) in 2021, which led to 923 hospitalizations and 196 deaths. The mortality rate in UE in 2021 was 13.7%, similar to that in 2020 (13.0%) and lower than in 2019 (17.6%) [26]. *L. monocytogenes* caused 23 outbreaks in UE in 2021, out of which 8 were classified as high-evidence outbreaks caused by: fish and fish products (4 cases), meat and meat products (3 cases), and broiler meat (1 case) [26]. In the United States, there were 4 identified outbreaks in 2021, caused by Queso Fresco (the outbreak caused 13 illnesses and 1 death), fully cooked chicken (3 illnesses, one death), and packed salads, which caused two independent outbreaks (10 illnesses and 1 death for one outbreak; 18 illnesses and 3 deaths for the second outbreak) [24].

### 1.3. Listeriosis and Antibiotics

CDC state that most people recover from intestinal illness without antibiotic treatment. Recommendations for individuals outside the high-risk groups include drinking extra fluids in cases when diarrhea occurs. However, antibiotics are needed for patients who have an invasive illness or a greater chance of becoming severely ill [27].

Among antibiotics used to treat listeriosis, ampicillin is typically the antibiotic of choice for human infections [28,29,30,31,32], interchangeably with amoxicillin, according to some sources [28]. Frequently gentamycin is recommended along with ampicillin as a treatment regimen due to its synergistic effects in vivo [28,29,30,31]. However, such a combination is not proven to improve patient survival rates [32]. A recent prospective observational cohort study has suggested that combined amoxicillin and gentamicin should be considered the first-line choice in invasive listeriosis [23]. In case of allergies to β-lactam antibiotics, when ampicillin cannot be administrated, trimethoprim with sulfamethoxazole is the generally recommended alternative to ampicillin [30,31,32]. Erythromycin and vancomycin are also listed as second-line antibiotics [29]. In animal infections, penicillin or oxytetracycline are recommended drugs [33].

### 1.4. Determination of Antibiotic Resistance in L. monocytogenes

The method typically applied by authors to determine antibiotic resistance of *L. monocytogenes* is disc diffusion [34,35,36,37,38,39,40,41,42,43], with differences in the procedures such as exact antibiotics used in the study, the content of the antibiotic in impregnated discs, divergent media, and varying incubation conditions. What is more, authors often decide to apply different criteria for interpreting the results of their experiments in terms of classifying particular strains as resistant, intermediate, or sensitive to antibiotics.

Clinical and Laboratory Standards Institute, Wayne, Pennsylvania state U.S. (CLSI) does not provide any standards for interpreting zones of inhibition achieved with the disc diffusion method of antibiotic susceptibility testing for *L. monocytogenes* [44]. The methodology recommended by CLSI for this organism includes the determination of Minimal Inhibitory Concentration (MIC) in cation-adjusted Mueller–Hinton broth supplemented with lysed horse blood (2.5–5% *v*/*v*). Given interpretive criteria include four antibiotics: penicillin, ampicillin, trimethoprim-sulfamethoxazole, and meropenem [45]. However, Bowker et al. presented results of the experiments suggesting that Mueller–Hinton agar with 5% defibrinated horse blood and 20 mg/L β-NAD (MH-F) is a suitable medium for antimicrobial susceptibility testing of *L. monocytogenes* for disk diffusion method, as authors have observed a correlation between MIC values and the zones of inhibitions [46]. Because of such observations, European Committee on Antimicrobial Susceptibility Testing, Basel, Switzerland (EUCAST) provides standards for interpreting zones of inhibition achieved in the disc diffusion method of antibiotic susceptibility testing of *L. monocytogenes*. Recommendations include using MH-F and incubation with 5% CO_2_ at 35 ± 1 °C for 18 ± 2 h. Criteria for interpreting the resistance status of isolates include five antibiotics: benzylpenicillin, ampicillin iv, meropenem, erythromycin, and trimethoprim-sulfamethoxazole at given disc contents [47].

Even though recent studies of antibiotic resistance of *L. monocytogenes* from food samples are available in the literature, the presented study will provide a positive addition to the information which is already published (even in cases when samples were collected in relatively close areas [41,42]), due to a continuous possibility of emerging changes in antimicrobial resistance patterns of microorganisms. What is more, every microbial collection is unique, and thus every study adds value to the bigger, global picture of antibiotic resistance. Due to these reasons, conducting studies on antibiotic resistance of pathogens repetitively by many researchers is justified and even necessary to monitor potential changes since unknown antimicrobial resistance patterns can be discovered in every study.

## 2. Materials and Methods

### 2.1. Bacterial Isolates

Bacterial isolates (*n* = 153) of *L. monocytogenes* originating from meat food samples (both raw and processed) and meat-processing environment (both being with contact and non-contacting with produced food) collected between October 2020 and November 2021 in Poland were used in the study. Species affiliation of collected isolates was confirmed with two methodologies, namely RFLP-PCR by Paillard et al. (2003) [48] and multiplex PCR by Li et al. (2021) [49]. The exact methodology of the sample collection and identification process is reported elsewhere [50]. Isolates preserved in Brain Heart Infusion (BHI) glycerol stocks stored at −80 °C constituted material for the study.

### 2.2. Antimicrobial Susceptibility Testing—Disc Diffusion Method

Isolates from glycerol stock were cultured on a BHI agar plate, incubated for 18 h, and subsequently stored at 4 °C for up to two weeks for further use. Control strains used in the study were *Escherichia coli* ATCC 25922 and *Staphylococcus aureus* ATCC 29213. Single colonies from the agar plate were used to inoculate 5 mL of liquid BHI medium (Oxoid) prewarmed to room temperature (20 ± 2 °C) and incubated at 37 °C for 18 h. Inoculated media were then centrifuged, and supernatants were discarded. Sediment was used to prepare 0.5 ± 0.05 McFarland standard suspension in sterile distilled water. A sterile cotton swab was immersed in the prepared solution and used to inoculate (by smearing in three directions) a petri dish with Mueller Hinton Agar (Oxoid). Discs impregnated with antibiotics (Oxoid) were then aseptically placed on inoculated plates (up to 5 discs on one plate) and incubated at 37 °C for 18 ± 2 h. Zones of inhibition were measured after incubation in opened plates and with reflected light and expressed in mm. The singular zone of inhibition was measured three times, and a mean value was calculated. Experiments were done in two separate technical repeats, from which mean values of the inhibition zones were calculated. Antibiotic discs used in the study included: ampicillin (AMP; 10 µg), chloramphenicol (C; 30 µg), ciprofloxacin (CIP; 5 µg), erythromycin (E; 15 µg), gentamicin (CN; 10 µg), penicillin (P; 10 IU), streptomycin (S; 10 µg), sulfamethoxazole/trimethoprim (SXT; 1.25/23.75 µg), tetracycline (TE; 30 µg) and vancomycin (VA; 30 µg). Zones of inhibition were interpreted according to CLSI standards [44] using the interpretive criteria for *Enterococcus* spp. when possible (in the case when the criteria were available for tested antibiotics) and Enterobacterales in case of remaining antibiotics: gentamycin, streptomycin, and sulfamethoxazole/trimethoprim. Microsoft Excel software was used to analyze and visualize the data.

### 2.3. Fingerprinting—RAPD- and REP-PCR

For selected isolates, RAPD- and REP-PCR fingerprinting techniques were used. RAPD-PCR was performed with OMP-01 primer (5′-GTTGGTGGCT-3′) in the reaction with a final concentration of 0.3 µM primer and 200 µM dNTP, along with 0.3 µL RUN polymerase (A&A Biotechnology, Gdańsk, Poland), compatible RUN buffer at suggested concentration, 1 µL of template DNA and water to achieve the final volume of 10 µL. The reaction temperature profile was as follows: (95 °C for 5 min, 35 °C for 2 min, 72 °C for 1 min) × 45, 72 °C for 10 min [51]. REP-PCR was performed with REP 1R-I (5′-IIIICGICGICATCIGGC-3′) and REP 2-I (5′-ICGICTTATCIGGCCTAC-3′) primers in the reaction with a final concentration of 1 µM of each primer, 200 µM dNTP, 250 µM MgCl_2_, along with 0.2 µL RUN polymerase, compatible RUN buffer at suggested concentration, 1 µL of template DNA and water to achieve the final volume of 10 µL. The reaction temperature profile was as follows: 95 °C for 5 min (90 °C for 30 s, 40 °C for 1 min, 72 °C for 1 min) × 30, 72 °C for 8 min [52]. PCR reactions were performed in a T-Gradient thermocycler (Biometra, Göttingen, Germany). Separation of amplified products was performed in 2100 Bioanalyzer Instrument (Agilent, Santa Clara, California State U.S.) using compatible DNA 12000 Kit (Agilent), according to manufacturer instructions.

The results achieved with both techniques in the form of generated gel images were connected together in order to create longer separation lines and provide more data. Analysis of aligned gels was performed with GelClust software, v.1.0.0.0 [53] with parameters set as sensitivity; 43, step; 8, error; 3, clustering method; UPGMA and distance coefficient; Jaccard. During the band marking step, manual adjustments were made if necessary.

## 3. Results

### 3.1. Antibiotic Susceptibility

Antibiotic susceptibility tests of 153 *L. monocytogenes* isolates (originating from meat products and processing environment) using the disc diffusion method were performed. Among ten antibiotics selected for this study, those commonly used to treat listeriosis were included. Results of performed experiments, along with applied interpretive criteria, are presented in Table 1.

Almost all (*n* = 143, 93.5%) collected bacteria were susceptible to all tested antibiotics. None of the isolates was resistant to more than one antibiotic. The only antibiotic to which collected isolates showed reduced susceptibility was ciprofloxacin (5 µg), to which ten isolates (6.5%) were not fully susceptible. However, according to applied criteria, they were not resistant either and were classified as intermediate.

**Figure 1:**
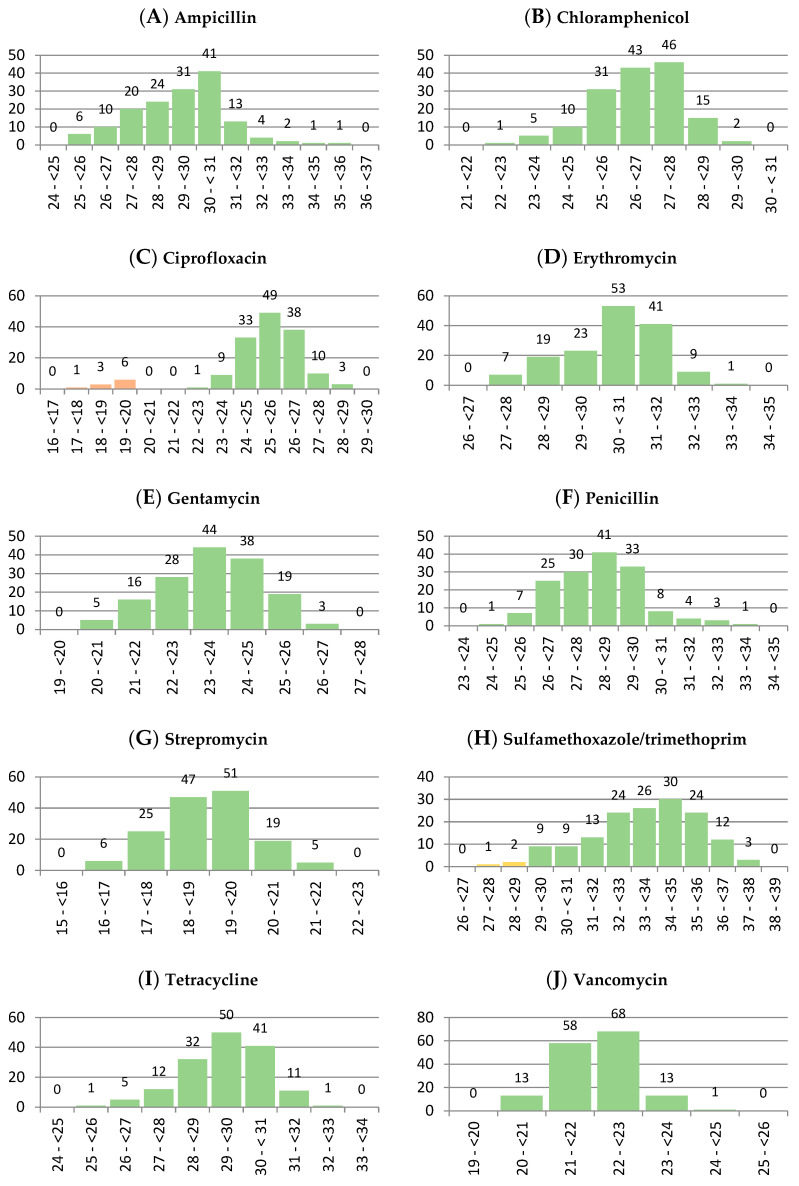
Histograms showing the distribution of inhibition zones (expressed in mm) of the *L. monocytogenes* isolates for the following antibiotics: (**A**): ampicillin (10 µg), (**B**): chloramphenicol (30 µg), (**C**): ciprofloxacin (5 µg), (**D**): erythromycin (15 µg), (**E**): gentamicin (10 µg), (**F**): penicillin (10 IU µg), (**G**): streptomycin (10 µg), (**H**): sulfamethoxazole/trimethoprim (1.25/23.75 µg), (**I**): tetracycline (30 µg), (**J**): vancomycin (30 µg).

Histograms showing the distribution of inhibition zones in the sample collection are presented in Figure 1. All tested antibiotics, with the exception of ciprofloxacin, present a unimodal distribution of zones of inhibition. In the case of ciprofloxacin, there is a visible non-symmetric bimodal distribution, with a subpopulation of samples (indicated as orange bars in Figure 1C) with reduced zones of inhibition. All ten isolates classified as intermediate in terms of their susceptibility to ciprofloxacin with predetermined criteria are also clearly separated from other samples on the histogram.

All of the ten isolates with reduced susceptibility to ciprofloxacin were collected from the surface swaps of the food-processing environment, including the meat injector (99B), transport box (81), ham wrapper (100A), exit door from the smoking chamber (45), hands of the worker weighting smoked mackerel fillets (in the smoked mackerel packing hall) (58B), gloves of the worker filleting the mackerel (98B), gloves of the worker working in smoking chambers area (67), swap from three boards (in the smoked mackerel packing hall) (96B), swap from four boxes (in the hall of cutting and packing smoked salmon) (65) and swap from four metal mats (in the hall of cutting and packing smoked salmon) (97B). The first three isolates were sampled on the same day, in the same processing plant, as well as the last seven isolates. All ten isolates present the same genoserotype VIb (representing serotypes 4b, 4d, and 4e), established with Doumith et al. (2004) protocol [54] (data not shown, see reference [50]).

### 3.2. Fingerprinting Results

**Figure 2:**
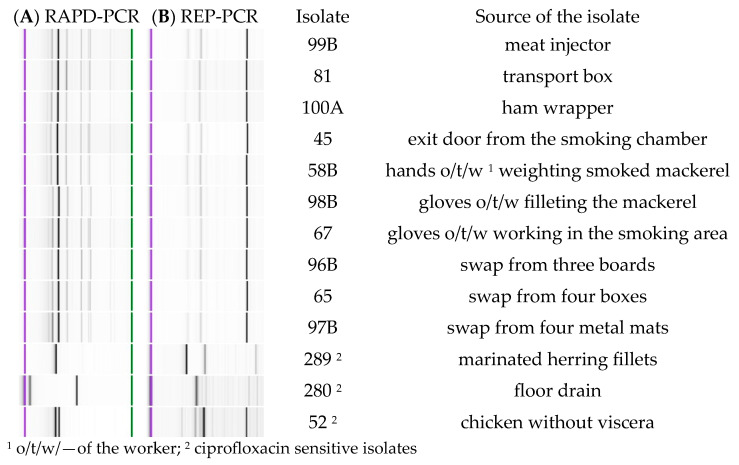
Patterns achieved with the fingerprinting methods (**A**): RAPD-PCR (**B**): REP-PCR.

RAPD- and REP-PCR fingerprinting of the ten isolates with reduced susceptibility to ciprofloxacin was performed. The patterns of the bands for those samples, as well as three other isolates sensitive to ciprofloxacin, are presented in Figure 2 below.

**Figure 3:**
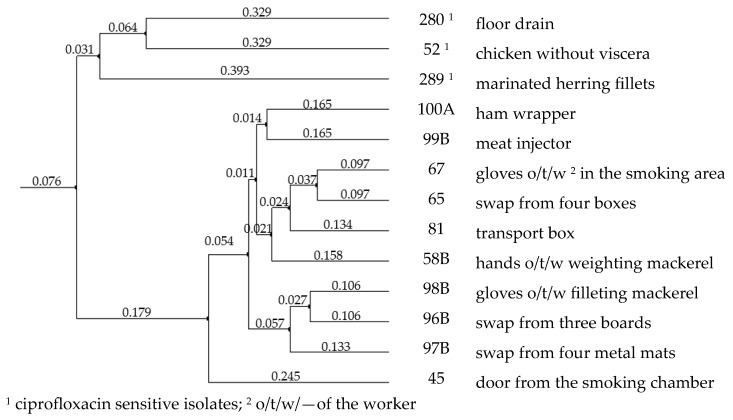
Phylogenetic tree generated based on aligned RAPD- and REP-PCR band patterns of the isolates.

Achieved band patterns for the ten isolates with reduced susceptibility to ciprofloxacin are very similar within both methodologies, sometimes with differences in the intensity of particular bands, which may be a measurement error (the Agilent 1200 Kit used in this study has the quantitation accuracy of 25%). All of the bands are discernible in all ten samples, with only slight differences in the product sizing, which also can be caused by a measurement error (the kit used provides ± 15% sizing accuracy). In comparison, patterns for three other isolates, which showed clear differences within their separation lanes, are also included. A phylogenetic tree created with GelClust software is presented in Figure 3.

In the phylogenetic tree, the ten samples with reduced susceptibility to ciprofloxacin are clustered together, with the distance coefficient within the sample group falling between 0.097 in the case of the most similar samples (which originated from a swap from four boxes and gloves of the worker working in smoking chambers area) and 0.245 in case of the samples which are the most distant within this cluster. On the other hand, the three isolates sensitive to ciprofloxacin are clearly separated from the cluster of samples with reduced susceptibility to the antibiotic. The distance coefficient between the two created clusters reached 0.424, which indicates that the samples intermediate to ciprofloxacin are more distinct from the sensitive samples than they are distinct from themselves within their cluster.

Based on these results, we conclude that all ten isolates with reduced ciprofloxacin sensitivity are probably a contamination of the processing facility, which occurred on food-contacting surfaces during processing.

## 4. Discussion

Authors apply different criteria to interpret the data achieved with the disc diffusion method when testing antibiotic resistance of *L. monocytogenes*. For example, Ebakota et al. (2018) decided to use the disc diffusion method using the Mueller–Hinton Agar medium and applied criteria as sensitive when the zone of inhibition reached >20 mm, intermediate in case of zones in a range of 15–19 mm and resistant when the zones were ≤14 mm (regardless of the antibiotic used) [40], whereas for example Maurice Bilung et al. (2018), who also used disc diffusion method on Mueller–Hinton agar plate, decided to apply CLSI criteria for staphylococci [34]. Some other authors state that they interpreted the values of zones of inhibition according to CLSI. However, the exact interpretation criteria are not given in the publications [37,39], whereas others apply methodology adapted from CLSI (with non-modified Mueller–Hinton agar medium) and decide to interpret the data in accordance with EUCAST guidelines (regardless of the fact that EUCAST recommends using MH-F medium) [41].

The chosen interpretative criteria applied herein were taken from CLSI guidelines, as a methodology closest to CLSI recommendations was also used in this study. Due to a lack of criteria for *L. monocytogenes*, zones of inhibition dedicated for *Enterococcus* spp. were used and completed with guidelines for Enterobacterales in cases where ranges for *Enterococcus* spp. were not provided. Predetermined criteria did not cause a separation of samples that had an appearance of one population in created histograms. What is more, a subpopulation of samples clearly distinguished on the histogram in the case of ciprofloxacin (Figure 1C) was also separated with criteria adapted from CLSI. Interestingly, in the case of erythromycin and trimethoprim/sulfamethoxazole, the EUCAST recommendations include using the same disc content of antibiotics (15 µg and 1.25/23.75 µg, respectively) [47] as used in this study. For those concentrations, CLSI provides ranges of zones of inhibition of quality control strains [44], which predetermine the choice of the disc content.

Although EUCAST methodology requires using a modified medium (Mueller–Hinton agar + 5% defibrinated horse blood and 20 mg/L β-NAD in contrast to Mueller–Hinton agar), as well as different incubation conditions (with 5% CO_2_ in contrast to ambient air), their criteria also seem to be interesting for interpreting the data achieved with the methodology used in this study. In case of applying those criteria (which are: 25 mm or more for erythromycin and 29 mm or more for trimethoprim/sulfamethoxazole for the isolate to be considered sensitive), the boundary between resistant and sensitive bacteria would be closer (compared to the criteria used herein) to zones of inhibition achieved in this study in case of erythromycin (the lowest zones of inhibition observed in this study are in the range of 27 to 28 mm), but the classification of the strains as sensitive would remain unchanged. However, three isolates (out of 153 tested) would have to be classified as resistant to trimethoprim/sulfamethoxazole (indicated as yellow bars in Figure 1H). Interestingly, two of these three isolates are also on the list of ten with reduced susceptibility to ciprofloxacin. These isolates originate from the hands of the workers weighting smoked mackerel fillets (in the smoked mackerel packing hall) (58B) and meat injector (99B), whereas the third one originates from Vienna type sausage (210) (and has a genoserotype IIa representing serotypes 1/2a, 3a [50]).

However, in the disc diffusion method, even a 1 mm difference in the measurement can result in a different interpretation of the tested isolate as either sensitive or resistant. One should be aware that the results of the experiment may vary, e.g., due to differences in diffusion of the antimicrobial agent in the media caused by variations of conditions such as water activity, not to mention the final measurement error itself. For example, in one study, 128 isolates of *L. monocytogenes* tested against erythromycin presented a range of zones of inhibition between 27 and 38 mm in one laboratory. However, the range widened to 21 to 39 mm when tested at five sites, even using the same methodology. Similarly, in the same study, 126 isolates of *L. monocytogenes* presented ranges of inhibition caused by trimethoprim/sulfamethoxazole varying from 18 to 38 mm in one laboratory with clear separation of the resistant subpopulation from the sensitive strains in the form of bimodal distribution on a histogram, whereas when tested at five laboratories, the distribution of the results widened to the range of 12 to 40 mm with no more clear separation of resistant and sensitive isolates on the histogram, but rather a skewed left distribution was achieved [46]. Even strains recommended by CLSI guidelines to perform quality control experiments are given wide zones of inhibition. For example, *Escherichia coli* ATCC 25922 should create a zone of inhibition ranging from 29 to 38 mm (which is more than 31% difference between the lower value and the upper value) when tested with ciprofloxacin, whereas other control strain, *Staphylococcus aureus* ATCC 25923, tested with the same antibiotic, should create a 22 to 30 mm zone (which is more than 36% difference between the readings of the extremes) [44] and both extreme values still indicate that the experiments are valid. Due to these factors, applying an intermediate range to interpretative criteria is, in our opinion, a justified attempt so that a small difference in a final measurement would not drastically affect the interpretation.

Due to the abovementioned limitations, other approaches in determining antibiotic resistance could be used, including the determination of the minimum inhibitory concentrations, e.g., by broth dilution methods [55,56]. However, the disc diffusion method is still recommended for testing many pathogens by institutions such as CLSI and EUCAST, and it is routinely applied in accredited laboratories worldwide, as well as is referred to in numerous scientific papers. Hence the general value of this methodology should not be neglected. 

In terms of comparing results achieved in this study to previous findings, in a recent paper published in 2022, 40 *L. monocytogenes* strains (isolated from food manufactured in Poland and Polish food processing environment) were tested for their sensitivity to 12 antibiotics, using disc diffusion method on Mueller–Hinton agar. Similarly to the results achieved herein, the authors also classified 100% of their samples as sensitive to ampicillin, chloramphenicol, erythromycin, tetracycline, and vancomycin. What is more, the authors identified two isolates (5%) with intermediate sensitivity to ciprofloxacin, which is a very similar rate to achieved herein (which is 6.5%). Five isolates (12.5%) were classified as resistant to trimethoprim/sulfamethoxazole, and one isolate (2.5%) was resistant to penicillin. In the case of gentamycin, one isolate (2.5%) was classified as intermediate [41].

In a different study (published in 2021) from the European Union, namely Italy, out of 98 *L. monocytogenes* samples originating from Italian slaughterhouses, processing plants, and fresh hams produced by these facilities, all isolates were susceptible to vancomycin, ampicillin, gentamicin, penicillin and streptomycin, which is in agreement with our findings. What is more, some of the isolates were also resistant to ciprofloxacin. However, the prevalence of such isolates was higher than in our publication and reached 42 samples (43%). Authors also identified isolates resistant to erythromycin (2 isolates), tetracycline (3 samples), and trimethoprim/sulfamethoxazole (3 samples) [42], whereas herein all isolates were susceptible to these antimicrobials.

In the study from Chile, published in 2022, all 14 tested *L. monocytogenes* isolates originating from various ready-to-eat food products were susceptible to chloramphenicol, erythromycin, penicillin, trimethoprim/sulfamethoxazole, tetracycline, and vancomycin [35], which is in agreement with our results. However, contrary to previously presented papers, all of the isolates were also classified as sensitive to ciprofloxacin. Interestingly, three isolates (21%) in the paper were classified as resistant to ampicillin, out of which two originated from plant-based foods, whereas the third one originated from cooked sausage [35]. The relatively high rate of ampicillin-resistant isolates is contrary to the results of our paper and is especially concerning given the fact that ampicillin is the antibiotic of choice in treating listeriosis.

The prevalence of resistant isolates was also higher than in our report in the case of a study published in 2021, which included 177 *L. monocytogenes* samples isolated in South Africa. Isolates originated from raw seafood, raw meats, and ready-to-eat foods, processing environment, and clinical samples. The authors determined the resistance of the isolates to 5 antibiotics, using the disc diffusion method on MH-F agar. Considering only 157 non-clinical samples included in the study, the authors identified 45 isolates (29%) resistant to erythromycin, 19 isolates (12%) resistant to tetracycline, 16 (10%) isolates resistant to chloramphenicol, and four isolates (3%) resistant to gentamycin [36], whereas all samples included in our study were sensitive to these antibiotics. Although different breakpoint zone criteria were used for interpreting the data in our paper, even a direct adaptation of the criteria from the discussed study would not have changed the results achieved by classifying isolates as sensitive in any case. On the other hand, similarly to our findings, all of the isolates from the discussed study (including clinical ones) were sensitive to ampicillin, which is an antibiotic of choice against *L. monocytogenes,* also in South African clinics and hospitals [36].

Contrary to our report and many abovementioned findings, all isolates (100%) from one study published in 2020 were resistant to ampicillin, as well as to penicillin. The research included 53 *L. monocytogenes* strains originating from imported beef cattle in Jordan. The authors used the disc diffusion method on Mueller–Hinton agar medium complemented with 5% defibrinated sheep blood to determine antibiotic resistance of the samples. Moreover, more than 90% of isolates were classified as resistant to clindamycin, tetracycline, and erythromycin, more than 80% were resistant to quinupristin/dalfopristin and linezolid, more than 70% showed resistance to streptomycin, teicoplanin, kanamycin, vancomycin, and ciprofloxacin, more than half of the isolates were resistant to ceftriaxone and gentamicin, as well as more than 40% of the isolates were resistant to chloramphenicol [43]. The prevalence of resistant and multi-drug resistant isolates is exceptionally high in this discussed paper. However, it is worth mentioning that for both ampicillin and penicillin, the author used ≤28 mm as a breakpoint criterium between sensitive and resistant bacteria (in the study, the same disc content of antibiotic was used as in our paper). Adapting these criteria to our study would lead to the reclassification of some isolates as resistant, namely 43 (28%) and 69 (45%) of isolates in cases of ampicillin and penicillin, respectively. However, in the case of chloramphenicol, gentamicin, erythromycin, streptomycin, tetracycline, and vancomycin (in every case, the same disc content of antibiotic was used herein and in discussed publication), a direct adaptation of the criteria set would not have affected the interpretation of the results. What is more, in the case of ciprofloxacin, the ten isolates that were considered intermediate would still be classified as sensitive.

Although there are many similarities between the results achieved in our paper and the studies from the same country (Poland) examining samples collected in a close timeframe [41], as well as from other European Union country (Italy) [42], in general, lower occurrence of antibiotic resistance was observed in our collection of *L. monocytogenes* isolates than in other presented studies. Additional fingerprinting analyses performed in our report showed similarities within the isolates with an intermediate response to ciprofloxacin. However, further investigation is needed to uncover whether the ten ciprofloxacin intermediate isolates are clones of the same origin or rather a genotype-specific for the ciprofloxacin-intermediate phenotype of *L. monocytogenes*.

## 5. Conclusions

*L. monocytogenes* isolates present different patterns of antibiotic resistance in different studies, depending on their source (in terms of types of products) as well as origin (in terms of geographical locations). Many similarities in the results can be observed in this study and in reports from a close area (the same country—Poland), as well as another European Union country (Italy), whereas papers from South Africa and Southwest Asia (Jordan) presented overall higher percentage of resistant isolates. Those area-dependent differences may originate from different practices of using antibiotics regionally (both in animal agriculture and in human medicine), as well as from existing regulations preventing the overuse of antibiotics in some areas. Applying different interpretative criteria to achieved results is also, to some extent, a contributor to differences in antimicrobial patterns presented in scientific papers.

Limitations of our study include shortcomings of the disc diffusion method itself, especially the fact that even a slight difference in the measurement of the zone of inhibition can result in different classifications of the tested isolate as either sensitive or resistant. 

The future perspective of our study includes additional investigation to disclose whether isolates with intermediate response to ciprofloxacin are clones of the same origin. More sensitive approaches than applied RAPD- and REP-PCR would include, for example, Pulsed Field Gel Electrophoresis or Whole Genome Sequencing, which would probably provide an answer to this question.

Improving global surveillance of drug resistance is one of the proposed solutions aiming to reduce the negative effects of antimicrobial resistance. Hence conducting research in this area repetitively by many scientists worldwide is crucial to monitor potential changes in antibiotic resistance patterns of pathogens globally.

## Figures and Tables

**Table 1:** Results of antibiotic susceptibility testing of *L. monocytogenes* isolates with disc diffusion method and interpretative criteria applied.

Antibiotic	Sensitive Isolates	Intermediate Isolates	Resistant Isolates
Ampicillin (10 µg)	≥17 mm	N/A ^1^	≤16 mm
	153 (100%)	-	0 (0%)
Chloramphenicol (30 µg)	≥18 mm	13–17 mm	≤12 mm
	153 (100%)	0 (0%)	0 (0%)
Ciprofloxacin (5 µg)	≥21 mm	16–20 mm	≤15 mm
	143 (93.5%)	10 (6.5%)	0 (0%)
Erythromycin (15 µg)	≥23 mm	14–22 mm	≤13 mm
	153 (100%)	0 (0%)	0 (0%)
Gentamicin (10 µg)	≥15 mm	13–14 mm	≤12 mm
	153 (100%)	0 (0%)	0 (0%)
Penicillin (10 IU µg)	≥15 mm	N/A	≤14 mm
	153 (100%)	-	0 (0%)
Streptomycin (10 µg)	≥15 mm	12–14 mm	≤11 mm
	153 (100%)	0 (0%)	0 (0%)
Sulfamethoxazole/trimethoprim (1.25/23.75 µg)	≥16 mm	11–15 mm	≤10 mm
	153 (100%)	0 (0%)	0 (0%)
Tetracycline (30 µg)	≥19 mm	15–18 mm	≤14 mm
	153 (100%)	0 (0%)	0 (0%)
Vancomycin (30 µg)	≥17 mm	15–16 mm	≤14 mm
	153 (100%)	0 (0%)	0 (0%)

^1^ Not apply.

## Data Availability

The data presented in this study are available on request from the corresponding authors.
